# Pharmacological Postconditioning with Lactic Acid and Hydrogen Rich Saline Alleviates Myocardial Reperfusion Injury in Rats

**DOI:** 10.1038/srep09858

**Published:** 2015-04-30

**Authors:** Guoming Zhang, Song Gao, Xiaoyan Li, Lulu Zhang, Hong Tan, Lin Xu, Yaoyu Chen, Yongjian Geng, Yanliang Lin, Benjamin Aertker, Yuanyuan Sun

**Affiliations:** 1 Department of Cardiology, the General Hospital of Jinan Military Command, Jinan 250031, China; 2 The Center of Cardiovascular Biology and Atherosclerosis Research, University of Texas Medical School at Houston, Houston, TX 77030, USA; 3 Department of Ultrasound, the General Hospital of Jinan Military Command, Jinan 250031, China; 4 Department of Hematology, School of Pharmacology, Nanjing Medical University, Nanjing, 210029, China; 5 Department of Center Laboratory, Provincial Hospital Affiliated to Shandong University, Jinan 250021, China

## Abstract

This study investigated whether pharmacological postconditioning with lactic acid and hydrogen rich saline can provide benefits similar to that of mechanical postconditioning. To our knowledge, this is the first therapeutic study to investigate the co-administration of lactic acid and hydrogen. SD rats were randomly divided into 6 groups: Sham, R/I, M-Post, Lac, Hyd, and Lac + Hyd. The left coronary artery was occluded for 45 min. Blood was withdrawn from the right atrium to measure pH. The rats were sacrificed at different time points to measure mitochondrial absorbance, infarct size, serum markers and apoptotic index. Rats in Lac + Hyd group had similar blood pH and ROS levels when compared to the M-Post group. Additionally, the infarct area was reduced to the same extent in Lac + Hyd and M-Post groups with a similar trends observed for serum markers of myocardial injury and apoptotic index. Although the level of P-ERK in Lac + Hyd group was lower, P-p38/JNK, TNFα, Caspase-8, mitochondrial absorbance and Cyt-c were all similar in Lac + Hyd and M-Post groups. The Lac and Hyd groups were able to partially mimic this protective role. These data suggested that pharmacological postconditioning with lactic acid and hydrogen rich saline nearly replicates the benefits of mechanical postconditioning.

Cardiovascular disease is currently one of the most common causes of mortality worldwide and in the United Sates^1^,^2^. Across the globe, the incidence of death from cardiovascular and circulatory diseases has increased over the past 10 years, accounting for nearly one in three deaths worldwide^3^,^4^. Of this total, acute myocardial infarction (AMI) is an important cause of death^5^. In the treatment of AMI, rapid and complete restoration of perfusion through emergent percutaneous coronary intervention or thrombolytic therapy has been widely used in clinical settings. However, this process may lead to severe reperfusion injury^6^. In 2003, Zhao *et* *al*. reported that mechanical postconditioning, consisting of brief intermittent cycles of ischemia alternating with reperfusion, after an ischemic event could ameliorate the effects of reperfusion injury^7^. Over the last several years increasing evidence from basic science and clinical studies have confirmed that mechanical postconditioning is an effective method for reducing reperfusion injury^8^,^9^,^10^,^11^,^12^.

However, the application of mechanical postconditioning is not without risk. Repeated distention of a catheter balloon in the lumen of arteries may result in plaque disruption and even distal embolization. In recent years, pharmacologic postconditioning has arisen as a potential alternative to mechanical postconditioning^13^,^14^. It is posited that the slow reintroduction of oxygen and persistence of tissue acidosis is the mechanism by which postconditioning provides benefit. Several studies have shown that acidic perfusion fluids recreate the protection provided by postconditioning, while reactive oxygen species (ROS) scavengers abrogate this effect^15^,^16^,^17^,^18^,^19^,^20^,^21^.

In this study, we investigated whether the combination of lactic acid and hydrogen rich saline produces the protective effects of postconditioning in a rat model of acute myocardial infarction. Lactic acid was chosen in an effort to better mimic the physiologic conditions of the tissue acidosis that develops following prolonged ischemia. Hydrogen was used due to its role as a ROS scavenger. Notably, hydrogen is able to selectively reduce the formation of hydroxyl radicals (• OH) and peroxynitirite anions (ONOO-), but it does not eliminate O^2−^ or H_2_O_2_. Peroxynitrite is a potent and versatile oxidant that can attack a wide range of biological molecules. It reacts with DNA, protein and lipids in physiologic conditions, resulting in cellular damage. Alternatively, O^2−^ and H_2_O_2 _are important signaling components in cardioprotection^22^,^23^,^24^,^25^,^26^. Therefore, the combination of lactic acid and hydrogen creates conditions which mimic the persistent tissue acidosis and allows for selective generation of ROS which are hypothesized to provide the beneficial effects seen with mechanical postconditioning.

## Results

### Hemodynamic parameters

**Figure 1:**
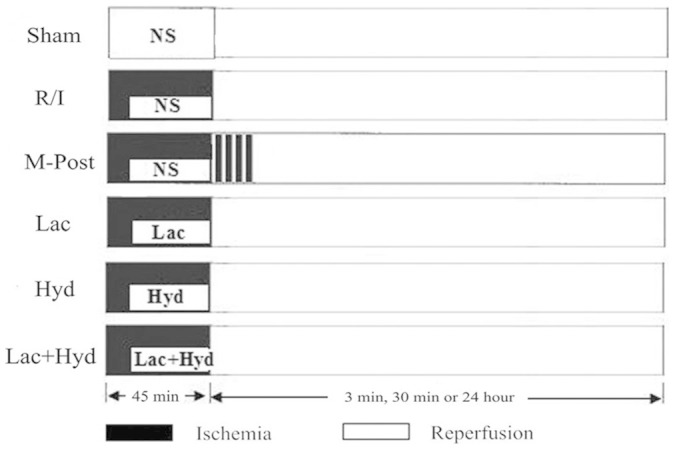
Timeline of the experimental protocols. After a 45 min period of acute myocardial infarction, the animals were injected with 0.9% normal saline (Sham, R/I and M-Post groups), whereas Lac, Hyd and Lac + Hyd group were, respectively, injected with lactic acid, hydrogen rich saline, and a combination of the two. Postconditioning consisted of 4, 20 s cycles of ischemia and reperfusion. Periods of reperfusion are shown in white and periods of ischemia are shown in black. Each group contained 18 rats.

The experiments were performed according to the protocols detailed in Fig. 1. Recordings of the hemodynamic parameters heart rate and mean arterial pressure (MAP) indicated that heart contractility was not affected in the various experimental groups following ischemia and reperfusion (Table 1). In contrast, there were changes in +dP/dt and −dP/dt. The value of +dP/dt and −dP/dt in M-Post, Lac, Hyd and Lac + Hyd groups were higher than in the R/I group (*P* < 0.05). Compared with the M-Post group, the measurements in the Lac and Hyd groups were significantly lower (*P* < 0.05). No significant difference was noted in the Lac + Hyd group (*P* > 0.05).

### pH value of right atrium after 3 min of reperfusion

After 3 min of reperfusion, the pH of the blood in the right atrium was measured to evaluate the impact of each treatment on the extension of local acidosis. As shown in Table 2, maintenance of acidosis was observed in M-Post, Lac, and Lac + Hyd groups as evidenced by significant decreases in blood pH (*P* < 0.05). No significant effect on blood pH was observed in the Hyd group (*P* > 0.05).

### Levels of MDA and SOD

Malondialdehyde (MDA) and superoxide dismutase (SOD) were used as indirect measures of ROS production. As shown in Table 2, M-Post, Hyd, and Lac + Hyd groups showed significantly reduced amounts of MDA and significant elevations in SOD activity (vs. R/I group; *P* < 0.05), with comparable efficiencies (*P* > 0.05). Lactic acid alone had no significant effect when compared to the R/I group (*P* > 0.05).

### Serum markers of cardiac damage

The R/I model caused significant increases in the intra-musclular levels of both CK and CK-MB (Table 2). All treatment groups showed significantly decreased levels of CK and CK-MB (vs. R/I group; *P* < 0.05). When compared with the M-Post group, no significant change was found in the Lac + Hyd group (*P* > 0.05). Levels of CK and CK-MB in the Lac and Hyd groups were significantly higher (*P* < 0.05).

### Infarct size

**Figure 2:**
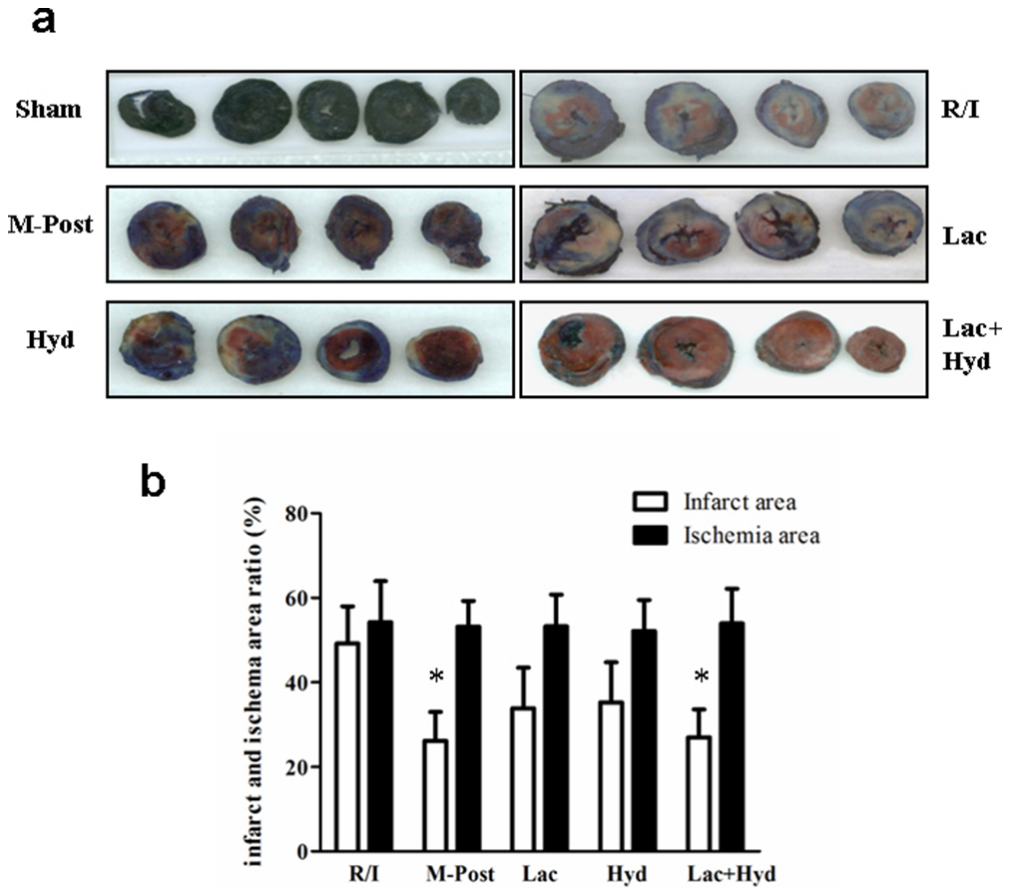
Analysis of ischemic and infarct area. (a) Ischemic (AAR/LV) and infarct (AN/AAR) areas for all groups; blue indicates a non-ischemic zone (dyed by Evans blue), red indicates an area of ischemia without infarction, and white indicates an infarct zone (dyed by triphenyltetrazolium chloride). (b) Ischemic (AAR/LV) and infarct (AN/AAR) sizes for all groups. The area of ischemia was similar among all groups, the infarct size of the Lac + Hyd group was significantly less than the R/I group (*P* < 0.05) and was similar to M-Post group (*P* *>* 0.05). Data represent means ± SD. *P < 0.05 vs. the R/I group.

Infarct size is an important index of myocardial injury because of its influence on heart contractility. As shown in Fig. 2, the ischemic area was similar among all the groups (52.17–54.25%). In contrast, the size of the infarct area in the M-Post and Lac + Hyd groups were significantly smaller than that in the R/I group (*P* < 0.05), but was similar between Lac + Hyd and M-Post groups (27.01 ± 6.56% vs 26.17 ± 6.85%, *P* > 0.05). In comparison to the R/I group, Lac and Hyd groups showed decreased infarct are size; however, the difference was not significant (*P* > 0.05).

### Cardiocytes apoptosis

**Figure 3:**
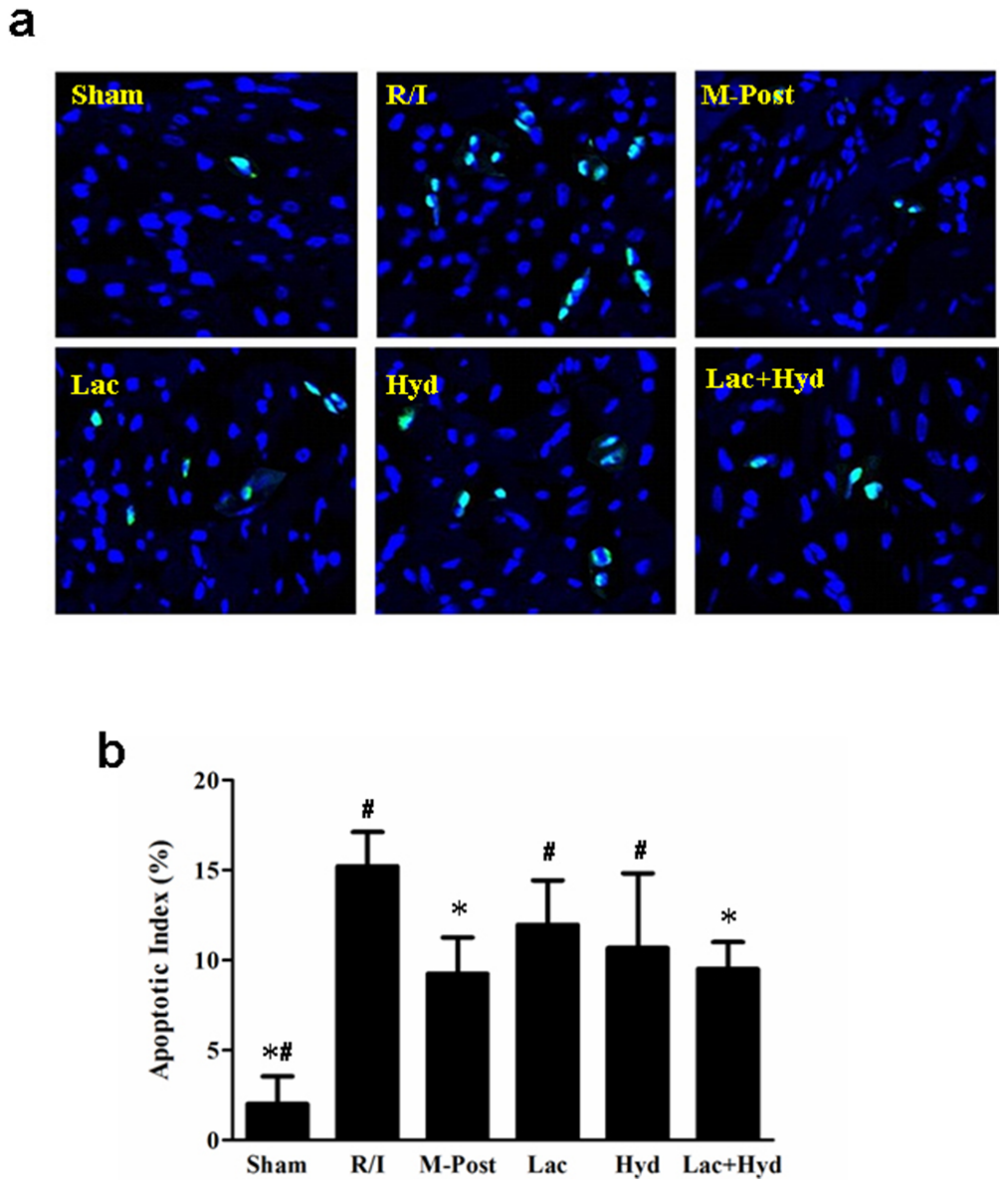
Detection of myocardial apoptosis. (a) Detection of apoptotic cells using TUNEL staining. TUNEL-positive cells were stained green. (b) Quantitative apoptotic index for all groups. Data represent means ± SD. *P < 0.05 vs. R/I group, ^#^ *P* < 0.05 vs. M-Post group.

The R/I model led to significant increase of apoptosis in cardiomyocytes (vs. Sham group, *P* < 0.05) as shown by the TUNEL assay (Fig. 3). The apoptotic indices of M-Post, Lac, Hyd, and Lac + Hyd groups were lower than that of the R/I group. The difference between the Lac + Hyd and R/I groups was also significant (9.51 ± 1.51% vs. 15.21 ± 1.91%, *P* < 0.05). The apoptotic index of Lac + Hyd group was similar to that of the M-Post group (9.51 ± 1.51% vs. 9.23 ± 2.03%, *P* > 0.05). The Lac and Hyd groups were significantly higher than that of the M-Post group (*P* < 0.05).

### Detection of the mPTP opening and Cyt-c expression in the cytosol

Mitochondrial absorbance is a marker of mitochondrial permeability transition pore (mPTP) opening. In this measurement, decreased absorbance correlates to increased mPTP opening. This indirect measure of mPTP opening was used to further elucidate the mechanism of action for pharmacologic postconditioning.

**Figure 4:**
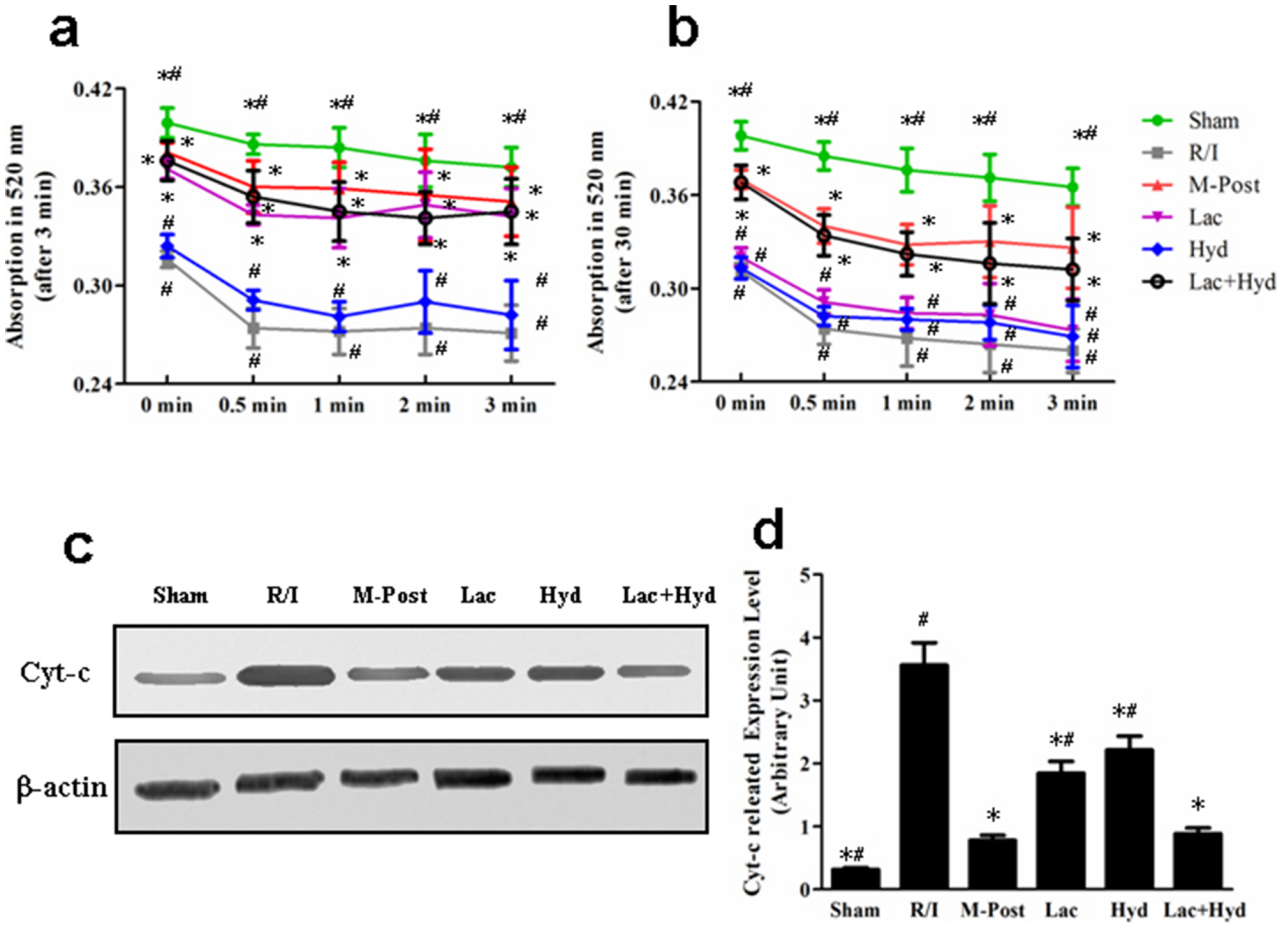
The measurement of mitochondrial absorbance after reperfusion and Cyt-c expression level in the cytosol. (a) The absorbance of mitochondria at 5 different time points following 3 min of reperfusion. (b) The absorbance of mitochondria following at 5 different time points following 30 min of reperfusion. (c) Western blot analysis of the expression levels of Cyt-c. (d) Densitometry of Western blot bands in the blots normalized by that of β-actin. The gels were run under the same experimental conditions. Data represent means ± SD. *P < 0.05 vs. the R/I group, ^#^ *P* < 0.05 vs. the M-Post group.

Myocardial mitochondrial absorbance was recorded at 5 discrete time points (0, 0.5, 1, 2 and 3 min) following 3 min and 30 min of reperfusion. As shown in Fig. 4a, after 3 min of reperfusion, the absorbance of all groups decreased following the initial measurement. The absorbance in the R/I group was the lowest, followed by Hyd, Lac, Lac + Hyd, and M-Post groups. The mitochondrial absorbance in Lac and Lac + Hyd groups were both significantly higher than for those in the R/I group (*P* < 0.05), and similar to the M-Post group (*P* > 0.05). In contrast, mitochondrial absorbance in the Hyd group was lower than in the M-Post group (*P* < 0.05), and similar to the R/I group (*P* > 0.05).

After 30 min of reperfusion (Fig. 4b), the mitochondrial absorbance in the Lac + Hyd group was also higher than in R/I group (*P* < 0.05) and similar to M-Post group (*P* > 0.05). The Hyd group remained lower than the M-Post group (*P* < 0.05) and similar to the R/I group (*P* > 0.05). The mitochondrial absorbance in the Lac group at 0 min was higher than that in the R/I group (*P* < 0.05), but there were no significant differences between Lac and R/I groups at 0.5, 1, 2 and 3 min (*P* > 0.05). Additionally, these values were significantly lower than those in the M-Post group at 1, 2 and 3 min (*P* < 0.05). These data showed that a solution consisting of hydrogen rich saline with lactic acid provides a longer duration during which mPTP is inhibited than is seen with lactic acid alone.

Cyt-c is an important pro-apoptotic factor which activates Caspase-9 and leads to apoptosis. To explore the possible involvement of this pathway in pharmacological postconditioning, we measured its expression. As shown in Fig. 4 c and d, the level of Cyt-c in M-Post, Lac, Hyd and Lac + Hyd group were significantly lower than in the R/I group (*P* <0.05). When compared with the M-Post group, no significant difference was seen with the Lac + Hyd group (*P* > 0.05). However, Cyt-c levels in Lac and Hyd groups were significantly higher (*P* <0.05) than in the M-post group.

### Impact of postconditioning on MAPK pathway

**Figure 5:**
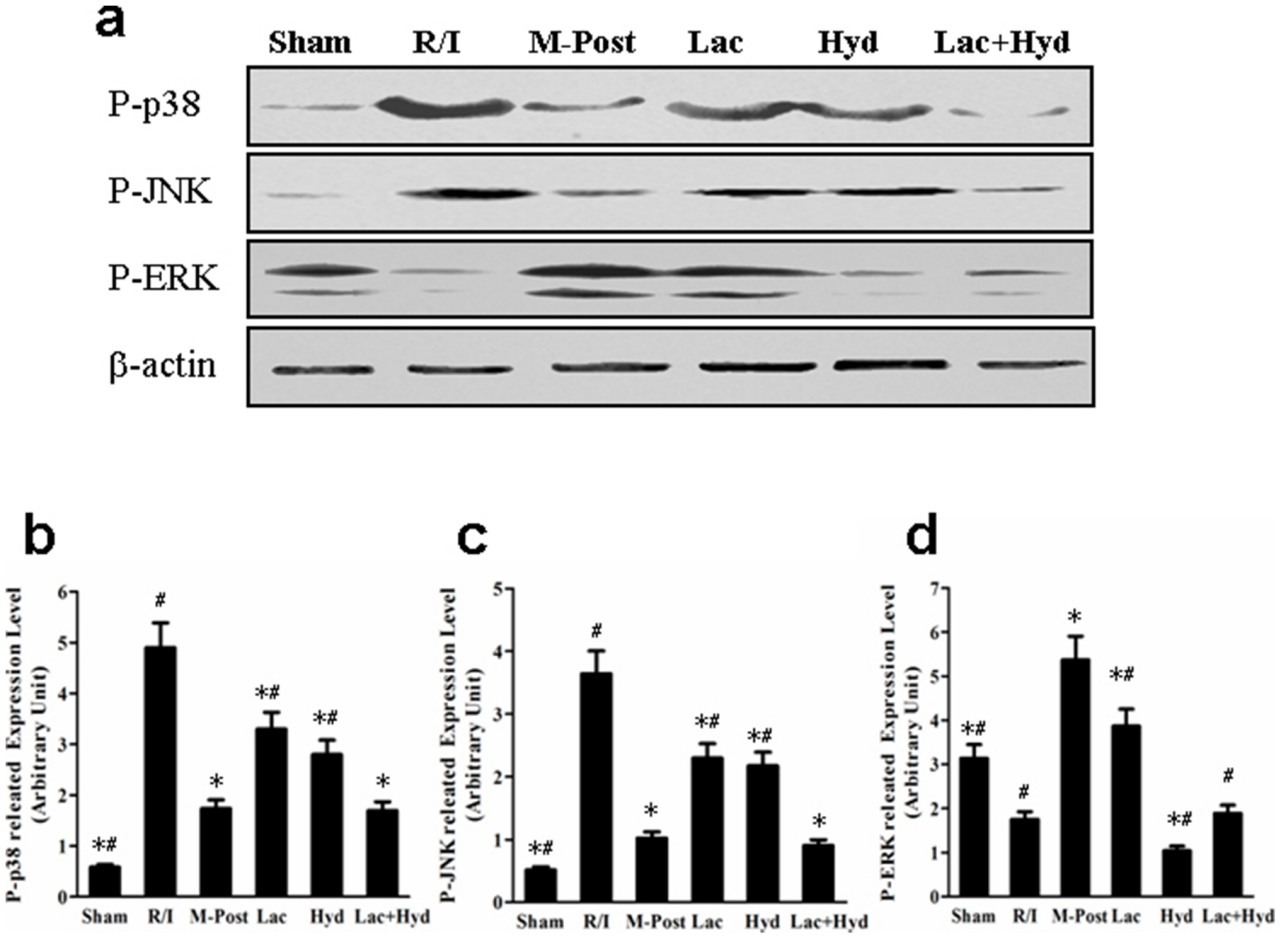
The phosphorylation expression of p38, JNK and ERK in MAPK signal pathway. (a) Western blot analysis of the phosphorylation expression of p38, JNK and ERK. (b) Densitometry of Western blot bands in the blots normalized by that of β-actin. The gels were run under the same experimental conditions. Data represent means ± SD. *P < 0.05 vs. the R/I group, ^#^ *P* < 0.05 vs. the M-Post group.

Postconditioning induces the phosphorylation of ERK, and inhibits the phosphorylation of p38 and JNK in the MAPK signal pathway^27^,^28^. To decipher the potential signaling pathways underlying pharmacological postconditioning of Lac and Hyd, we measured the phosphorylation of key proteins in MAPK pathway. The levels of P-p38 and P-JNK were significantly decreased in M-Post, Lac, Hyd and Lac + Hyd groups when compared with the R/I group (*P* < 0.05) (see Fig 5 a, b and c). These levels in the Lac and Hyd groups were significantly higher than those detected in the M-Post group (*P* < 0.05).

When compared with the R/I group, the level of P-ERK showed completely different changes (Fig. 5 a and d). The level of P-ERK in Lac and M-Post groups was significantly higher when compared to the R/I group (*P* < 0.05). Alternatively, a reduction in P-ERK was observed in the Hyd group (*P* < 0.05), and the Lac + Hyd group remained similar to the R/I group (*P* > 0.05). The expression of P-ERK in the Lac, Hyd, and Lac + Hyd groups was significantly lower than that of the M-Post group (*P* < 0.05).

**Figure 6:**
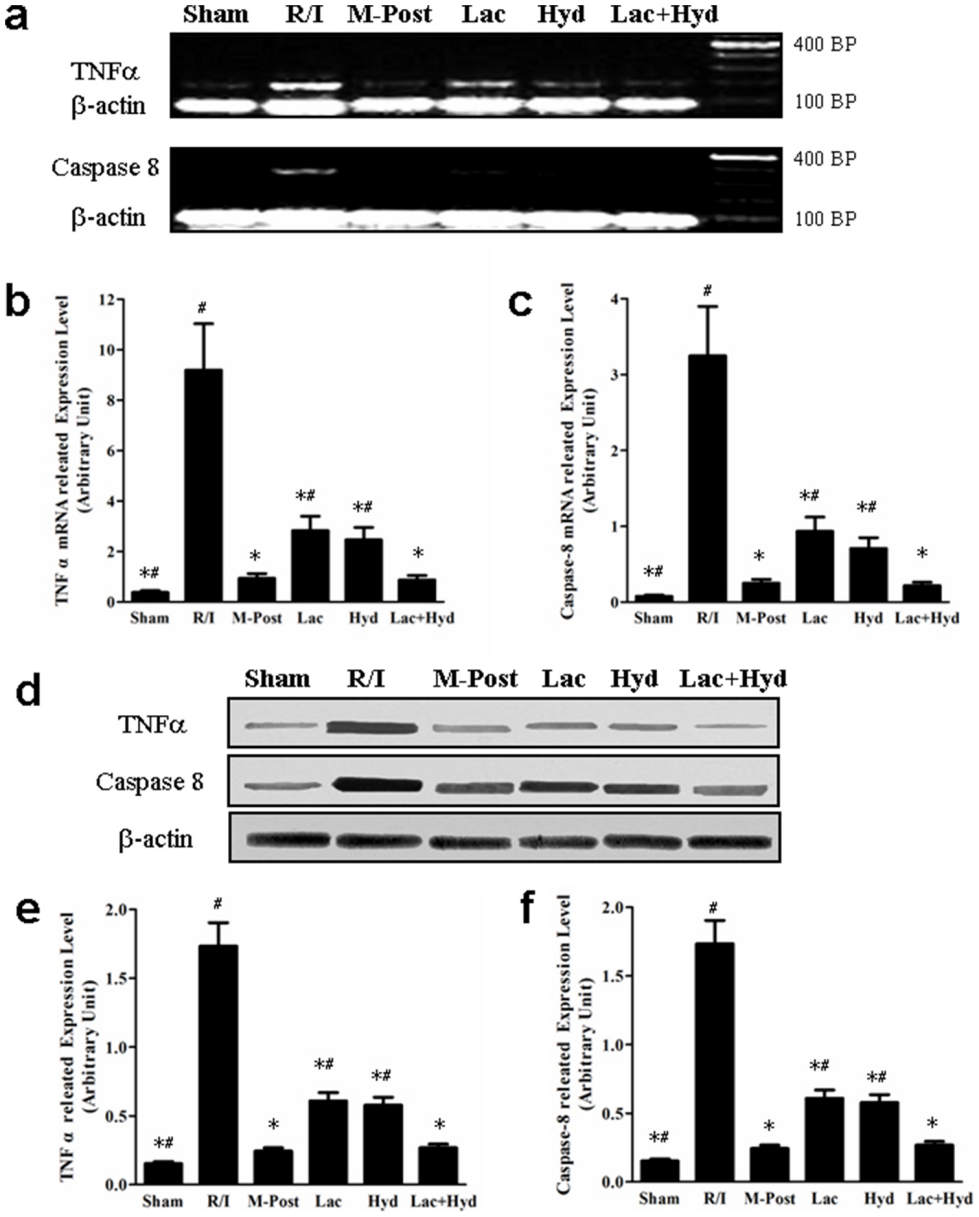
The expression of MAPK signal pathway downstream molecules. (a) RT-PCR analysis of TNFα and Caspase-8 in total RNA extracted from tissue. (b) Quantitative real-time RT-PCR analysis of TNFα and Caspase-8 in total RNA extracted from tissue. (c) Western blot analysis of the expression levels of TNFα and Caspase-8. (d) Densitometry of Western blot bands in the blots normalized by that of β-actin. The gels were run under the same experimental conditions. Data represent means ± SD. *P < 0.05 vs. the R/I group, ^#^ *P* < 0.05 vs. the M-Post group.

The expression of two downstream molecules of the MAPK pathway, TNFa and Caspase-8, were also measured due to their involvement in apoptosis and necrosis^29^,^30^,^31^. Protein and mRNA expression of TNFα and Caspase-8 showed similar trends (Fig. 6). The levels of TNFα and Caspase-8 in M-Post, Lac, Hyd, and Lac + Hyd groups were all significantly lower than that of the R/I group (*P* < 0.05). When compared to the M-Post group, the expression of these proteins was similar in the Lac + Hyd group (*P* > 0.05), but was significantly higher in Lac and Hyd groups (*P* < 0.05).

## Discussion

Reperfusion is a critical treatment for minimizing myocardial damage following AMI. However, reperfusion can lead to new injury due to cell death by both apoptosis and necrosis. Postconditioning provides a means to alleviate the extent of reperfusion injury. In light of the potential limitations and pitfalls seen with mechanical adaptation, pharmacological postconditioning provides another strategy for reducing reperfusion injury. Prior studies have demonstrated that drugs which have been shown to reduce infarct size may do so in part through their activation of the RISK pathway, and ultimately inhibition of mPTP opening^32^,^33^. However, these agents are not believed to mimic all of the upstream factors associated with mechanical postconditioning^34^.

Short-term persistence of local tissue acidosis and delayed recovery of local oxygen levels may be important mediators of postconditioning. The use of hydrogen rich saline with lactic acid was chosen to provide an alternative method of achieving some of the benefits seen with mechanical postconditioning. As lactic acid is the main component of the tissue acidosis seen following AMI, its inclusion allowed for a closer approximation of the physiologic conditions seen following ischemia^15^,^16^,^17^,^18^,^19^,^20^,^21^. Furthermore, hydrogen rich saline was evaluated due to its potential for protection by selectively inhibiting of ROS generation^22^,^35^,^36^ without influencing the pH of the ischemic tissue^37^,^38^.

This study demonstrated that treatment with lactatic acid resulted in lower pH levels following ischemia that approximated that of animals undergoing mechanical postconditioning. Additionally, the level of ROS, as shown indirectly through measurements of MDA and SOD activity, in the Hyd group was comparable to that of the M-Post group. Treatment with lactic acid was able to extend the period of acidosis in ischemic tissue, but did not significantly influence the generation of ROS. In this study, a trend toward a reduction in ROS was also observed; Nonetheless, this difference was not significant. However, other investigators have reported that lactic acid may reduce ROS^39^,^40^. This discrepancy may be due to the use of a surrogate marker for ROS production as opposed to more direct measurements of ROS. These data indicate that the combination of hydrogen rich saline and lactic acid provide a way to achieve the degree of acidosis and ROS generation that are hypothesized to trigger postconditioning.

The cumulative effect of myocardial tissue injury through apoptosis and necrosis may be observed in hemodynamic changes. In our study, animals treated with hydrogen rich saline with lactic acid showed nearly the same degree of cardioprotection as those undergoing mechanical postconditioning. However, the Lac and Hyd groups in this study only provided a portion of the cardioprotection demonstrated by the M-Post group. Consistent with previous studies using hydrogen gas or hydrogen rich saline alone^36^,^41^, our results support the advantage of co-administration of lactic acid and hydrogen rich saline, and further verify the acidosis hypothesis of postconditioning.

The mPTP consists of a collection of protein complexes between the inner and outer mitochondrial membranes and plays an important role in apoptosis by allowing the passive diffusion of solutes to occur^42^,^43^. In this study, mitochondrial absorbance and Cyt-c levels were used to evaluate the opening of mPTP^44^. Our results showed that a combination of lactic acid and hydrogen rich saline was able to play an inhibitory role on mPTP opening at 3 and 30 min following reperfusion. Treatment with lactic acid alone appeared to provide inhibition only over the shorter interval and no effect at either time point was found with hydrogen rich saline alone. These results appear to show that the protection mediated by postconditioning is dependent on both the prolongation of acidic conditions and alteration of the amount and type of ROS produced^45^,^46^.

A deleterious effect of ROS on mitochondria is also displayed in a model of age-related skeletal muscle dysfunction. Umanskaya et al demonstrated a decrease in the pathologic intracellular Ca leak with antioxidant treatment. This intracellular finding was associated with gross changes in anatomical function in the form of whole organism exercise capacity and the specific force performance of skeletal muscle^47^. These findings further strengthen the position that ROS are involved in multiple pathophysiologic processes and the potential for antioxidant treatments that provide a selective yet adequate effect.

Although similar effects on apoptotic cell death, ROS generation, tissue pH and infarct size were observed, exploration of the MAPK pathway revealed that there may be differences in the upstream effects of mechanical postconditioning and hydrogen rich saline with lactic acid. Mechanical postconditioning increased the phosphorylation of ERK and inhibited the phosphorylation of p38 and JNK. The results of this experiment showed that hydrogen rich saline decreased the phosphorylation of both p38/JNK and ERK which is consistent with earlier studies^48^,^49^. Hydrogen rich saline alone also inhibited the expression of P-p38/JNK, but it did not inhibit the expression of TNFα and Caspase-8 or provide the same degree of cardioprotection as mechanical as seen with mechanical postconditioning. Despite the incongruent effects on P-ERK demonstrated by mechanical postconditioning and co-administration of lactic acid and hydrogen rich saline, the latter still resulted in inhibition of TNFα and Caspase-8 expression. Nearly the same degree of cardioprotection was seen with this combination when compared to mechanical postconditioning as well. This phenomenon might be attributable to the regulation of P-ERK by other unknown pathways involved in postconditioning.

Several limitations exist in this study. First, the pH of right atrial blood was used instead of direct measurement of the pH in the myocardial tissue. A second limitation was the use of MDA and SOD activity in lieu of directly measuring ROS generation. As discussed previously, this may account for the inability of this study to replicate prior works showing decreased ROS production with lactic acid treatment. Ideally, a direct measurement of the various ROS, including •OH,ONOO-, O^2−^or H_2_O_2, _would be performed. Future experiments should evaluate the use of multiple dose regimens for lactic acid and hydrogen rich saline. Further evaluation of other signaling molecules is also necessary to better characterize the intricate postconditioning process.

In summary, the combination of lactic acid and hydrogen rich saline initiates protective signaling pathways and replicates the cardioprotective role of mechanical postconditioning in spite of divergent effects on ERK phosphorylation. Further investigation may allow for the addition of this regimen to the armamentarium available for combatting the morbidity associated with AMI.

## Methods

### Animal

All animal experiments were performed in accordance with the guidelines set forth in the Guide for the Care and Use of Laboratory Animals (US National Institutes of Health, NIH) and was approved by the Research Commission on Ethics of the General Hospital of Jinan Military Command.

108 Sprague-Dawley (SD) rats, male and female, weighing 200–250 g (8 weeks old) were bought from Shandong University (Jinan,China). Rats were fed a standard rat chow diet (Vital River Laboratory Animal Technology Co. Beijing), and housed at 22°C with a 12-h light-dark cycle in an on-site animal facility.

### Rat heart model of acute myocardial infarction and later treatment

Rats were anesthetized with sodium pentobarbital (46 mg/kg, intraperitoneal injection) and were then intubated and ventilated using a rodent respirator (ventilation rate: 52 breaths per minute, tidal volume: 4.0 mL/100 g body weight). The left pleural cavity was opened, and a 5–0 ligature was placed under the left coronary artery (LCA) by inserting the thread into the left atrium and threading it out from the side of the pulmonary artery cone. A balloon (Grip, 3.0a12 mm, Acrostak Corp., Switzerland) connected to a pump full of water at a pressure of 1 atmosphere (ATM) was placed into the artery to achieve fast reperfusion and ischemia. After securing a knot, the pressure of the balloon was immediately adjusted to 12 ATM for 45 minutes of ischemia. Following the experimental protocols below, after 45 minutes occlusion, the pressure of the balloon was quickly adjusted to zero for various time periods (Fig. 1).

### Experimental protocols

Hydrogen rich saline was freshly prepared and used as previously described^36^. The pH of the normal saline solution (0.9% NaCl, NS) and hydrogen rich saline were adjusted to 7.4 by using 4 mol/L NaOH. The pH of the lactic acid solution (98%, Beijing Huibao Union-Chemistry Technology, Beijing, China) was adjusted to 5.5 by using 4 mol/L NaOH.

With the exception of the Sham group, rats were subjected to a 45-min acute myocardial infarction followed by reperfusion (Fig 1). At the onset of reperfusion the various solutions were injected into the myocardial tissue around the infarct zone through a micro-injector. According to the randomized and controlled principle, rats were divided into the 6 groups: (1) animals in the sham group underwent thoracotomy and NS injection (60 µL), without any other ischemia treatment; (2) the reperfusion-injury group (R/I) received NS (60 µL) and routine continuous reperfusion treatment; (3) the mechanical postconditioning group (M-Post) received NS (60 µL) and postconditioning treatment which consisted of 4 × 20 s cycles of alternating reperfusion/re-occlusion, and initiated immediately upon the onset of reperfusion; (4) the lactic acid group (Lac) received lactic acid (60 µL) and routine ischemia-reperfusion treatment; (5) the hydrogen rich saline group (Hyd) received hydrogen rich saline (60 µL) and routine ischemia-reperfusion treatment; (6) the lactic acid and hydrogen rich saline group (Lac + Hyd) received a combination of lactic acid (60 µL) and hydrogen rich saline (60 µL) followed by routine ischemia-reperfusion treatment.

3 minutes following reperfusion, right atrial blood (0.5 mL) was extracted through a microinjector to measure the pH using a blood gas analyzer. A sterile cotton ball was placed into the pleural cavity for 3 min to stop any bleeding.

To collect the various samples, animals were sacrificed at 3 min, 30 min and 24 h following reperfusion. The hearts were excised, according to the procedures described below (the anterior wall tissues of the left ventricles were kept at −80°C until used), to enable the following measurements: 1) the levels of malondialdehyde (MDA) and superoxide dismutase (SOD) activity were measured after 3 min of reperfusion; 2) mitochondrial swelling was measured to indirectly evaluate the mitochondrial permeability transition pore (mPTP) opening after 3 and 30 min of reperfusion; 3) levels of molecules involved in postconditioning signal pathways, such as MAPK, tissue necrosis factor (TNFα), Caspase-8 and cytochrome c (Cyt-c) were conducted following 30 min of reperfusion; 4) the infarct size was measured after 24 h of reperfusion, the hearts were dyed immediately as follow; 5) the apoptotic index was measured after 24 h of reperfusion, after soaking the myocardial tissue in 10% formalin (pH = 7.4).

### Hemodynamic parameters

After an initial intraperitoneal injection of urethane (2 g/kg), the right carotid artery was cannulated using an arterial catheter connected with a physiograph through a three-way stopcock. The heart rate and arterial pressure were physiographically monitored through the arterial catheter. Then +dp/dt and −dp/dt were analyzed using the physiograph, and the rate-pressure product (RPP) was calculated as the product of the rate and the mean arterial pressure.

### Levels of MDA and SOD activity in myocardial tissue

The left ventricular myocardium was homogenized^50^, and the levels of MDA and SOD activity were measured using a commercial kit (Lipid Peroxidation Assay Kit, Nanjing, China).

### Serum markers of myocardial muscle injury

Blood was withdrawn from the rats after 24 h of reperfusion and the sample was immediately centrifuged (1409 g × 10 min). Serum levels of creatine kinase (CK) and the MB isoenzyme of creatine kinase (CK-MB) were analyzed using an automatic biochemistry analyzer (Automatic Clinical Chemistry Analyser, BPC Biosed, Rome, Italy).

### Detection of apoptotic cells

Apoptotic cells were detected on transverse sections of the left ventricle using a DNA fragmentation detection kit (Roche Corp., Germany) based on the terminal deoxynucleotidyl transferase-mediated UTP nick end labeling (TUNEL) method. Apoptotic index was used to quantification above results: the number of positively stained apoptotic cardiocytes/total number of cardiocytes counted ×100%.

### Measurement of the infarct size

The infarct size was detected as described by Kerendi et al.^51^ The area of ischemic myocardium at risk (AAR), area of necrosis (AN), and left ventricle (LV) area were determined by area analysis using Image-Proplus software (version 4.1, Media Cybernetics, Rockville, MD, USA). The infarct size was expressed as AN/AAR, and the ischemic area was expressed as AAR/LV.

### Detection of mitochondrial permeability transition pore (mPTP) opening

Mitochondrial absorbance was measured as an indirtect measure of mPTP opening. The cytosolic and mitochondrial fractions were isolated using Mitochondrial Extraction and Absorbance Detection Kit ( Shanghai Genmed, Shanghai, China) (17). Mitochondria (200 µg protein) were diluted and treated with CaCl_2_ (150 umol/L) to induce opening of mPTP. Absorbance at 0, 0.5, 1, 2, and 3 min was recorded at 520 nm using a microplate reader (POTENOV Co., Beijing).

### Western blot and RT-PCR analysis of signaling molecules

The cellular responses to postconditioning are regulated by the MAPK signaling pathway and ultimately affect the mPTP^52^. Reverse transcription polymerase chain reaction (RT-PCR) and quantitative real-time RT-PCR (qRT-PCR) analysis of TNFα, Caspase-8 and β-actin mRNA was performed with TNFα, Caspase-8 and β-actin specific primers (Sigma Aldrich, St. Louis, MO), iQ SYBR Green Supermix kits (Bio-rad, Hercules, CA) and One-Step RT-PCR Kit (Qiagen, Valencia, CA). The relative mRNA abundance was determined following normalization to β-actin mRNA expression levels. RT-PCR products were analyzed by agarose gel electrophoresis followed by ethidium bromide staining. Western blot analyses were conducted to detect the impact of the reperfusion injury protection protocols on the pathway and the release of Cyt-c from the mitochondria^50^. Phospho-MAPK (P-ERK, p38 and pJNK), TNFα, Caspase-8, Cyt-c and β-actin specific antibodies were used (Santa Cruz Biotechnology, Santa Cruz, CA, USA). Antigen–antibody complexes were visualized by enhanced chemiluminescence (ECL Western Blotting Detection, BestBio, Shanghai, China).

### Statistical analysis

All values are reported as means ± SD. Data were analyzed using SPSS 13.0 for Windows statistical software package (IBM, Armonk, NY, USA). Differences were evaluated by Student’s *t* test or One-way analysis of variance (ANOVA). A two-tailed *P* < 0.05 was taken to indicate a statistically significant difference.

## Author Contributions

G.Z. designed and conducted the experiments, interpreted the data and drafted the manuscript. S.G. designed the experiments and drafted the manuscript. X.L. participated in the collection and interpretation of the Western blot analysis. L.Z. helped with the design of the experiments and to draft the manuscript. H.T. performed experiments and helped to draft the manuscript. L.X. helped with the design of the study, collected and analyzed data. Y.C. and Y.G. participated in design of animal studies and helped to draft the manuscript. Y.L. coordinated and interpreted molecular studies. B.A. helped to revise the manuscript. Y.S. coordinated and interpreted animal studies and participated in drafting the manuscript. All authors reviewed and approved the final manuscript.

## Supplementary Material

Supplementary Information Dataset 1

## Figures and Tables

**Table 1:** Hemodynamic parameters of each group after AMI and reperfusion

Group	HR (bpm)	MAP (mmHg)	+DP/DT (mmHg/s)	-DP/DT (mmHg/s)
Sham	398.50 ± 20.46*	106.62 ± 5.42	969.12 ± 60.61* #	679.81 ± 116.32* #
R/I	352.09 ± 18.18	99.07 ± 10.12	602.81 ± 61.74#	442.05 ± 37.71#
M-Post	375.98 ± 45.21	104.96 ± 4.99	782.61 ± 55.71*	563.65 ± 51.28*
Lac	356.31 ± 33.62	99.32± 7.35	685.61 ± 62.06* #	502.11 ± 41.46* #
Hyd	371.99 ± 39.28	98.69 ± 8.89	706.21 ± 63.61* #	523.12 ± 38.16* #
Lac + Hyd	374.12 ± 29.76	105.26 ± 3.71	779.12 ± 68.72*	558.17 ±42.25*

HR, heart rate; bpm, beats per minute; MAP, mean arterial pressure.*P < 0.05 vs. R/I, ^#^*P* < 0.05 vs. M-Post. (n = 6)

**Table 2:** pH, the level of MDA, SOD, and serum markers of cardiac damage

Group	pH	MDA (nmol/mgpro)	SOD (U/mgpro)	CK (U/L)	CK-MB (U/L)
Sham	7.45 ± 0.02#	0.82 ± 0.11#	75.78 ± 4.65* #	160.12 ± 33.43* #	98.15 ± 54.46* #
R/I	7.43 ± 0.02#	1.69 ± 0.21#	32.21 ± 12.41#	1340.52 ± 211.53#	969.81 ± 264.82#
M-Post	7.32 ± 0.02*	1.11 ± 0.32*	57.52 ± 13.12*	582.51 ± 263.16*	481.12 ± 152.93*
Lac	7.31 ± 0.06*	1.56 ± 0.28#	43.92 ± 18.71	879.01 ± 252.74* #	691.04 ± 328.65* #
Hyd	7.43 ± 0.04	1.14 ± 0.12*	55.92 ± 13.21*	754.23 ± 201.36* #	564.21 ± 173.22* *#
Lac + Hyd	7.33 ± 0.03*	1.13 ± 0.11*	56.47 ± 16.26*	649.02 ± 214.61*	475.35 ± 267.61*

MDA, malondialdehyde; SOD, superoxide dismutase; CK, creatine kinase; CK-MB, MB isoenzyme of creatine kinase; *P < 0.05 vs. R/I; ^#^*P* < 0.05 vs. M-Post. (pH: n = 18, MDA, SOD, CK and CK-MB: n = 6)
