# Supplementary material for: Pharmacological Postconditioning with Lactic Acid and Hydrogen Rich Saline Alleviates Myocardial Reperfusion Injury in Rats
Source: Sci Rep. 2015 Apr 30;5:9858. doi: 10.1038/srep09858 (PMC4415575; doi:10.1038/srep09858)
Supplement: Supplementary Information — Dataset 1 [file srep09858-s1.doc]

**Supplementary section to:**

**Pharmacological Postconditioning with Lactic Acid and Hydrogen Rich Saline Alleviates Myocardial Reperfusion Injury in Rats**

Guoming Zhang ^a,†^, Song Gao ^b, †^, Xiaoyan Li ^a^ , Lulu Zhang ^b^ , Hong Tan ^a^,*, Lin Xu ^a^, Yaoyu Chen ^d^, Yongjian Geng ^b^, Yanliang Lin ^e^, Benjamin Aertker ^b^, Yuanyuan Sun ^c^, *

^a^ Department of Cardiology, the General Hospital of Jinan Military Command, Jinan 250031, China.

^b^ The Center of Cardiovascular Biology and Atherosclerosis Research, University of Texas Medical School at Houston, Houston, TX 77030, USA.

^c^ Department of Ultrasound, the General Hospital of Jinan Military Command, Jinan 250031, China.

^d^ Department of Hematology, School of Pharmacology, Nanjing Medical University, Nanjing, 210029, China.

^e^ Department of Center Laboratory, Provincial Hospital Affiliated to Shandong University, Jinan 250021, China.

**^†^** These authors contributed equally to this work.

**Uncropped blots/images**


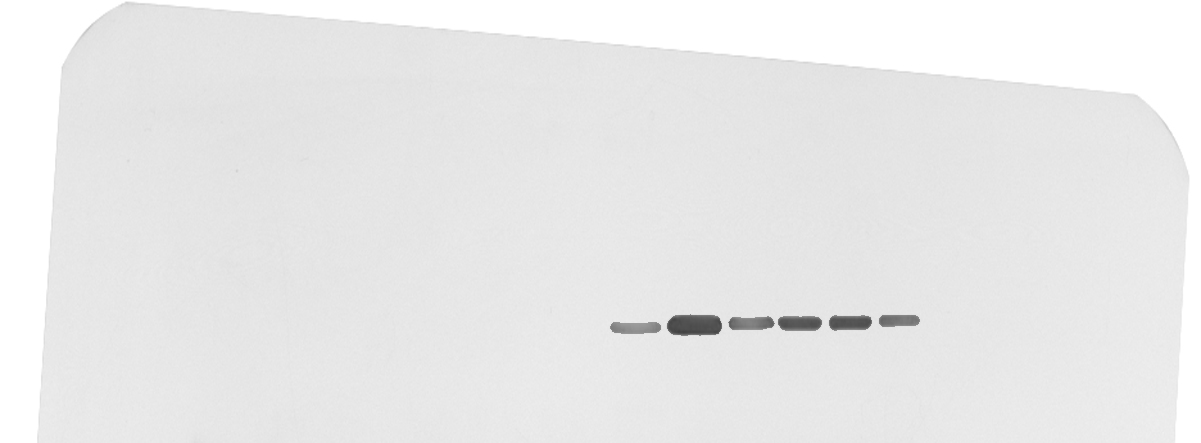


12 kD


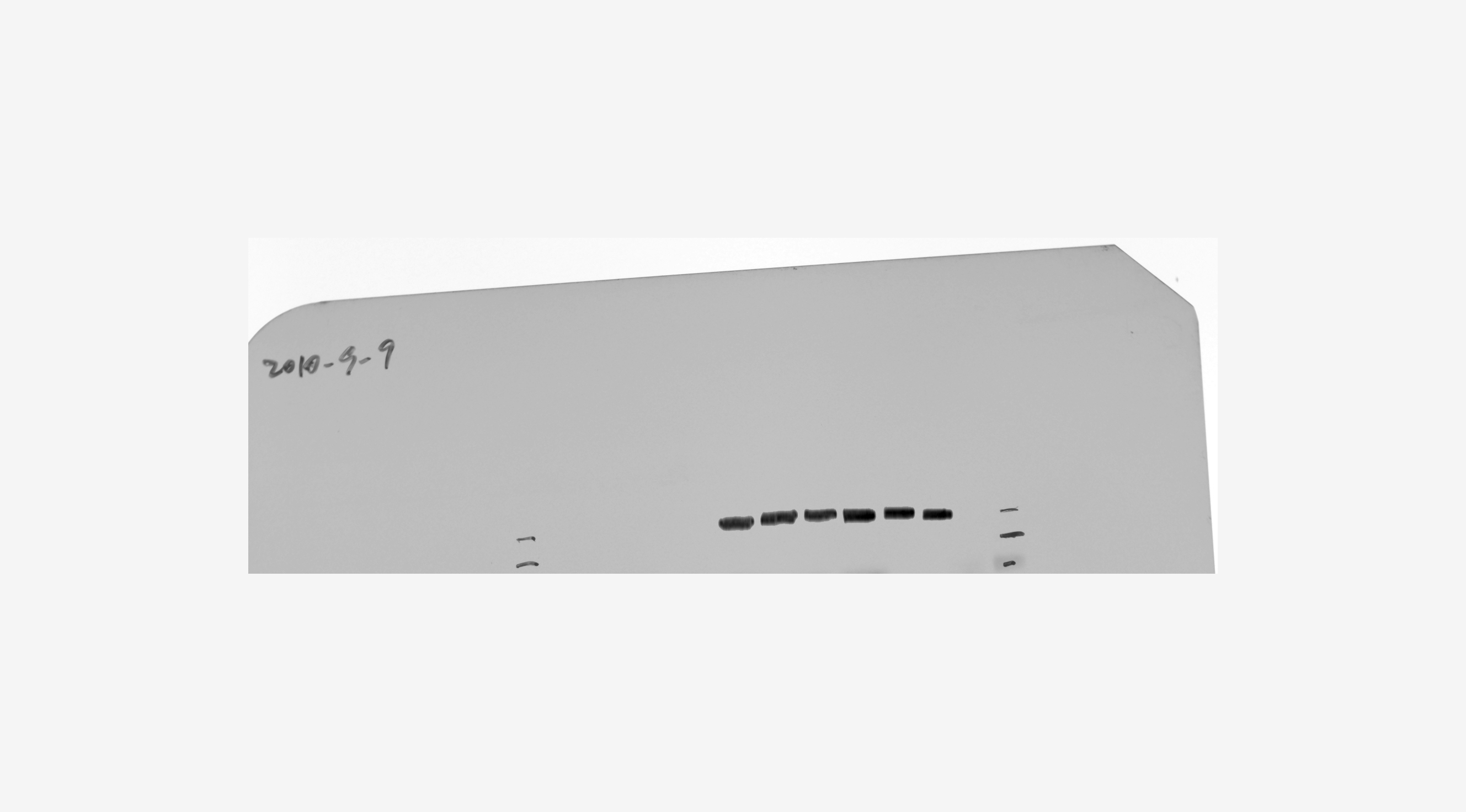


Cyt-c

42 kD

**Figure 4 C**

β-actin


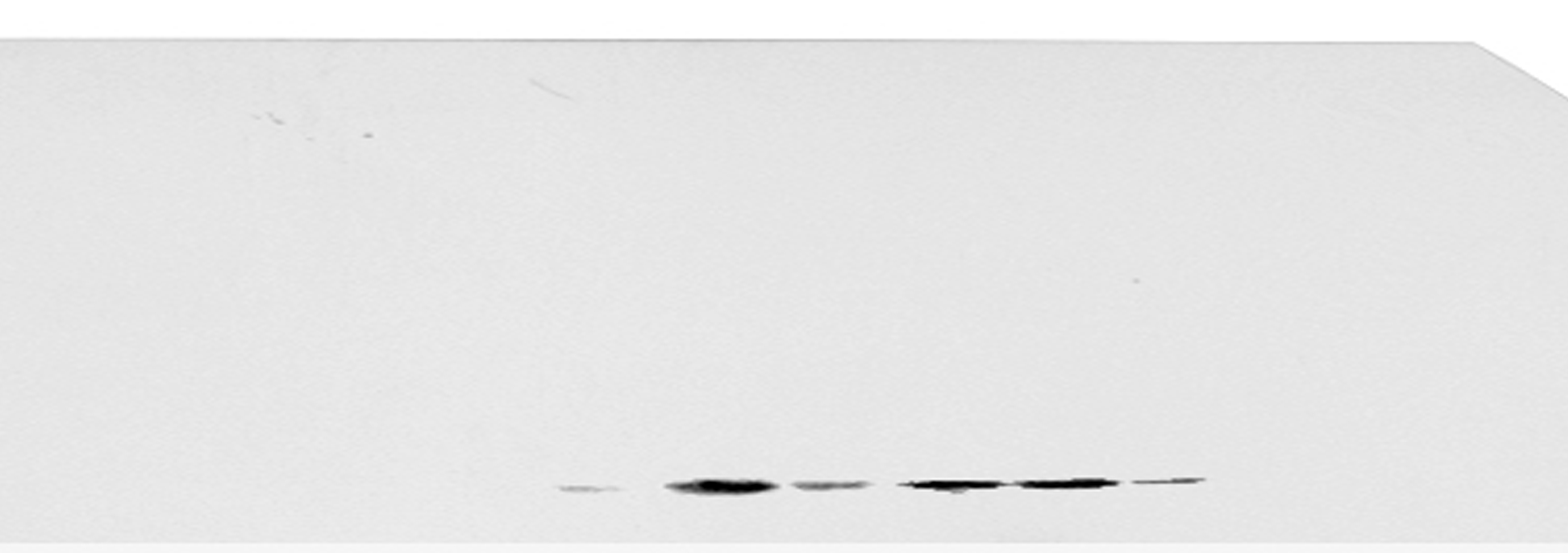

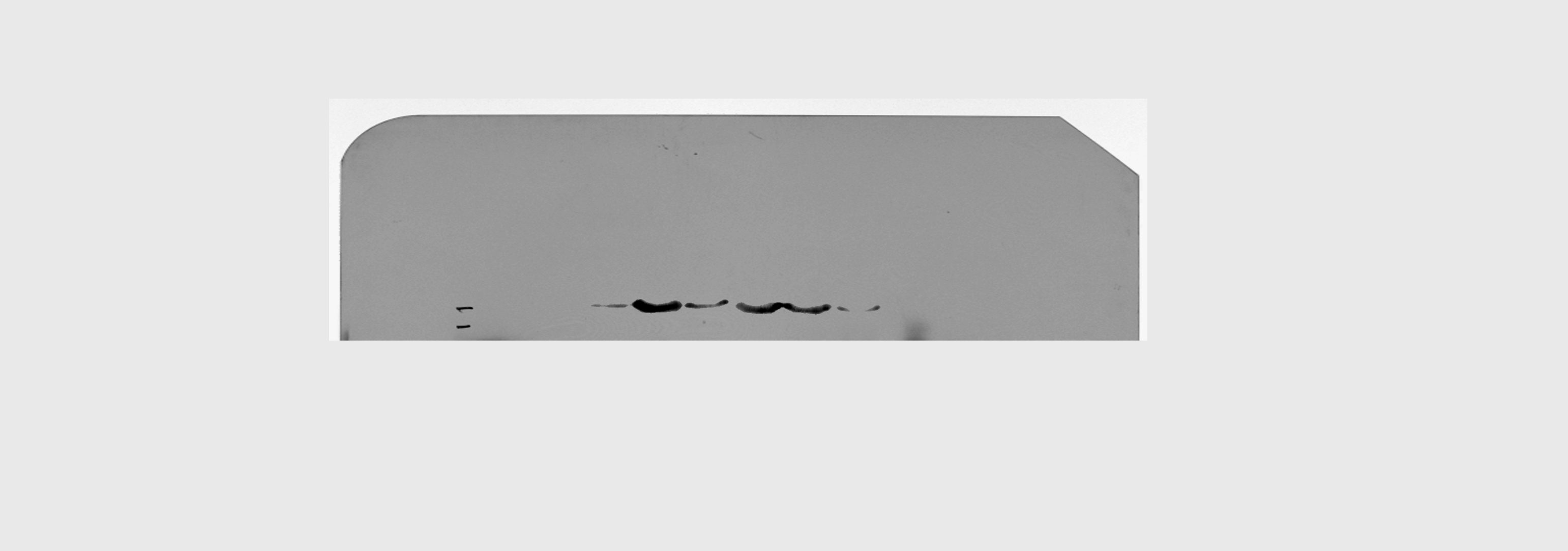


38 kD

P-p38

P-JNK

46 kD


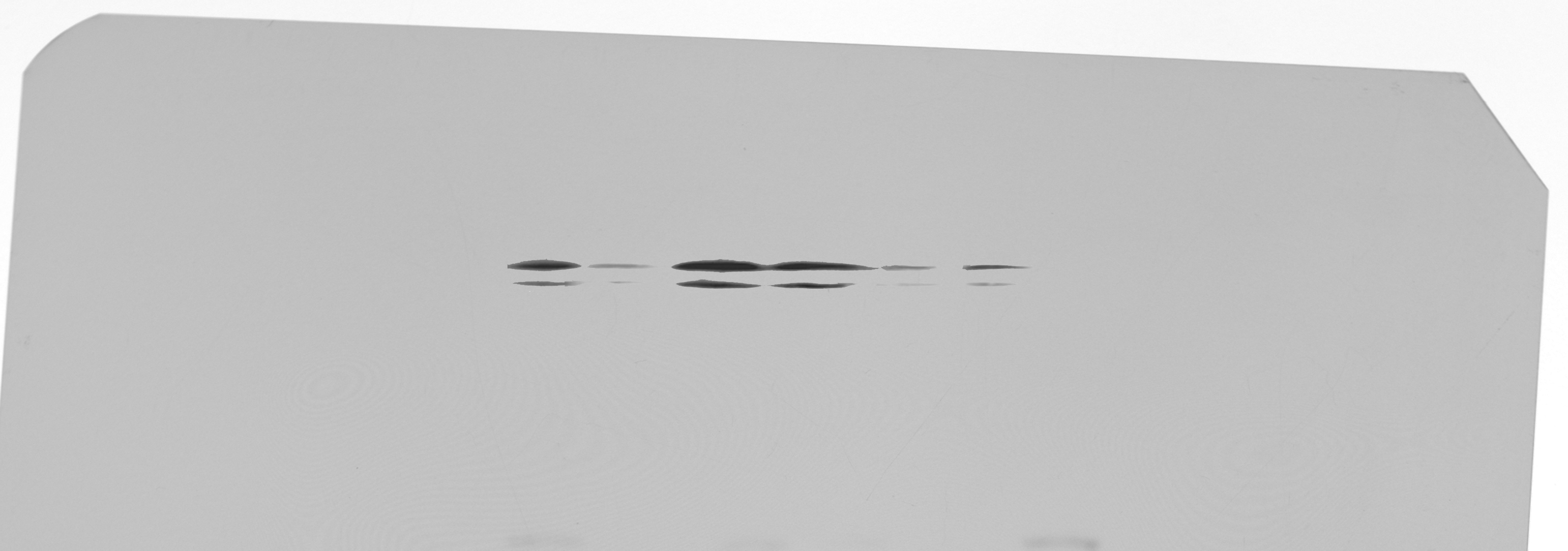


44 kD

42 kD

P-ERK


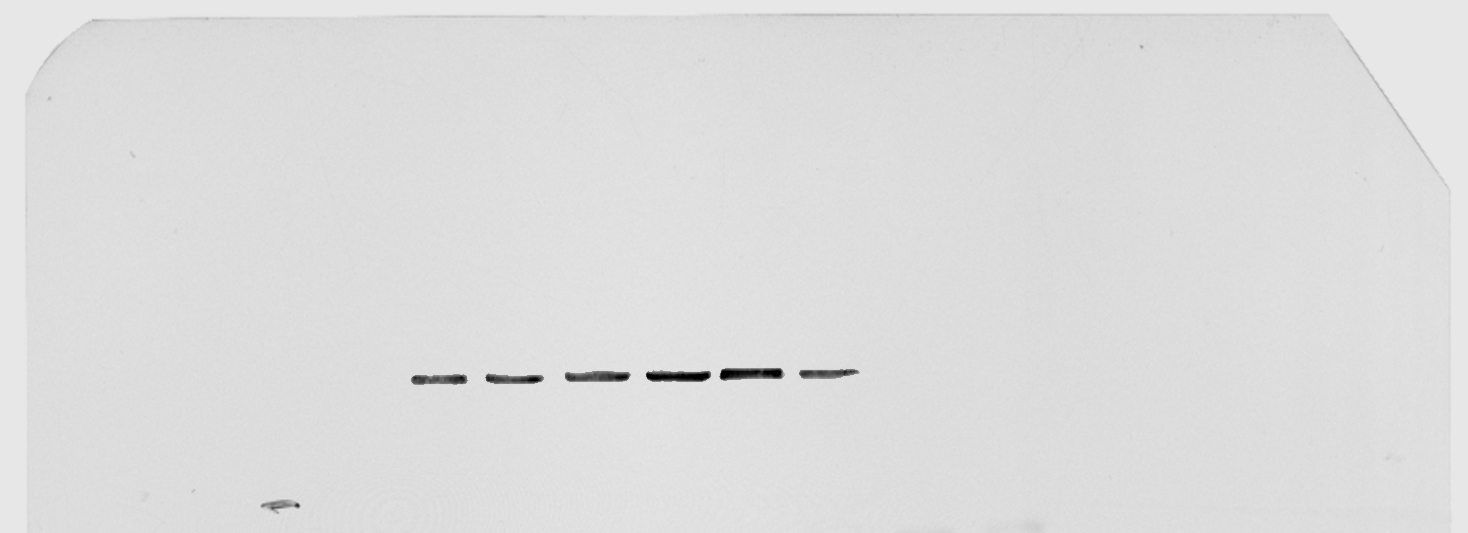


β-actin

42 kD

**Figure 5 A**


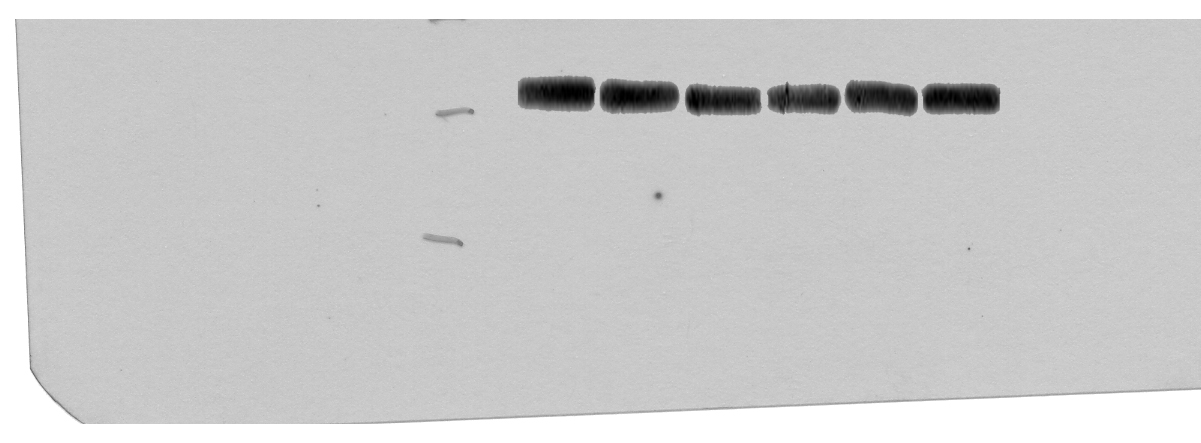


β-actin

42 kD


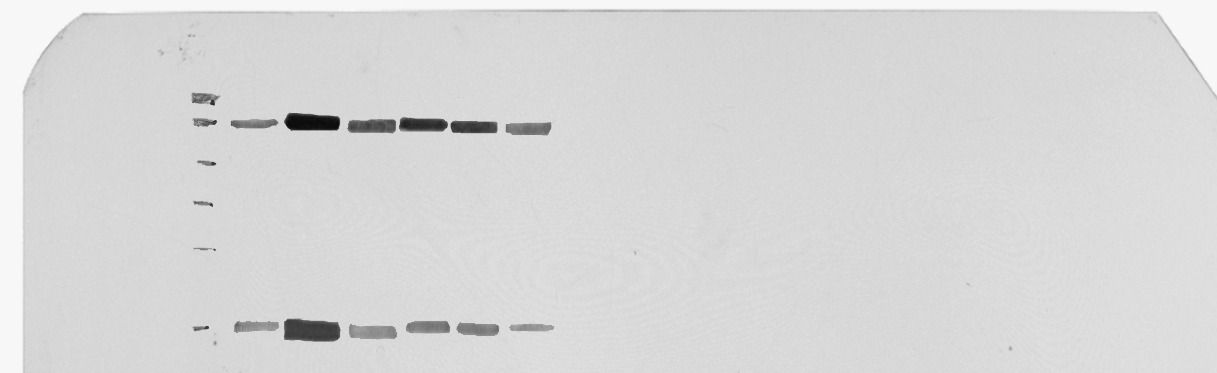


55 kD

17 kD

Caspase 8

TNFα

**Figure 6 A**


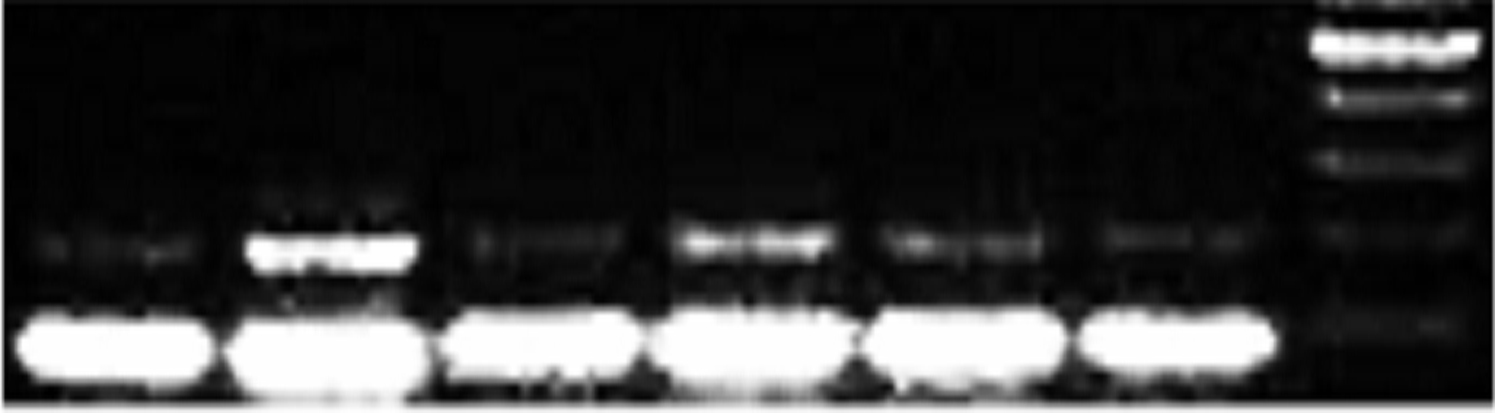


TNFα

Caspase 8

400 BP

100 BP


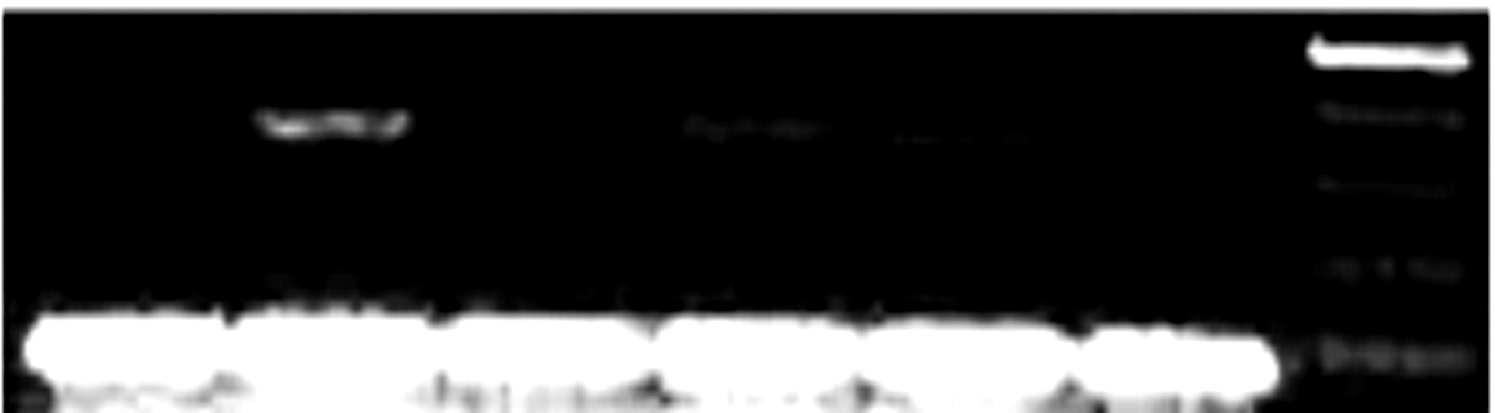


β-actin

β-actin

400 BP

100 BP

**Figure 6 D**
